# Contribution of Arab countries to pharmaceutical wastewater literature: a bibliometric and comparative analysis of research output

**DOI:** 10.1186/s40557-016-0117-0

**Published:** 2016-07-03

**Authors:** Sa’ed H. Zyoud, Shaher H. Zyoud, Samah W. Al-Jabi, Waleed M. Sweileh, Rahmat Awang

**Affiliations:** Poison Control and Drug Information Center (PCDIC), College of Medicine and Health Sciences, An-Najah National University, Nablus, 44839 Palestine; Department of Clinical and Community Pharmacy, College of Medicine and Health Sciences, An-Najah National University, Nablus, 44839 Palestine; WHO Collaborating Centre for Drug Information, National Poison Centre, Universiti Sains Malaysia (USM), Pulau Pinang, Penang 11800 Malaysia; Institute of Urban Water Management and Landscape Water Engineering, Technical University of Graz, Graz, Austria; Department of Pharmacology and Toxicology, College of Medicine and Health Sciences, An-Najah National University, Nablus, 44839 Palestine

**Keywords:** Arab world, Pharmaceutical wastewater, Bibliometric, Scopus, Non-Arab Middle Eastern countries

## Abstract

**Background:**

Recently, the pharmaceutical manufacturing industry has been growing rapidly in many countries in the world, including in Arab countries. Pharmaceuticals reach aquatic environments and are prevalent at small concentrations in wastewater from the drug manufacturing industry and hospitals. Such presence also occurs in domestic wastewater and results from the disposal of unused and expired medicines. Therefore, the objective of this study was to analyze and compare the quantity and quality of publications made by researchers in Arab countries on pharmaceutical wastewater.

**Methods:**

To retrieve documents related to pharmaceutical wastewater, we used the Scopus database on November 21, 2015. All documents with terms related to pharmaceutical wastewater in the title or abstract were analysed. Results obtained from Arab countries were compared with those obtained from Turkey, Iran and Israel.

**Results:**

Globally, a total of 6360 publications were retrieved while those from Arab countries, Iran, Turkey and Israel, were 179, 113, 96 and 54 publications respectively. The highest share of publications belonged to Kingdom of Saudi Arabia (KSA) with a total of 47 (26.2 %) publications, followed by Egypt (38; 21.2 %), Tunisia (17; 9.5 %) and Morocco (16; 8.9 %). The total number of citations was 1635, with a mean of 9.13 and a median (inter quartile range) of 3 (1.0–10.0). The study identified 87 (48.6 %) documents with 32 countries of international collaboration with Arab countries. It was noted that Arab researchers collaborated mainly with authors in Western Europe (54; 30.2 %), followed by authors from the Asiatic region (29; 16.2 %) and Northern America (15; 8.4 %). The most productive institution was *King Saud University*, KSA (13; 7.3 %), followed by the *National Research Centre*, Egypt (10; 7.3 %).

**Conclusions:**

This study showed that KSA has the largest share of productivity on pharmaceutical wastewater research. Bibliometric analysis demonstrated that research productivity, mainly from Arab countries in pharmaceutical wastewater research, was relatively lagging behind. More research effort is required for Arab countries to catch up with those of non-Arab Middle Easter countries on pharmaceutical wastewater research.

## Background

Pharmaceuticals reach aquatic environments and are prevalent at small concentrations in wastewaters from the drug manufacturing industry, hospital wastewaters, domestic wastewaters and due to the disposal of unused and expired medicines [1–4]. Many medications are disposed of without being completely metabolized in bodies, leading to biologically active forms mixing with water bodies [5–7], which can potentially affect drinking water supplies and human and ecosystem health [8].

Recently, the pharmaceutical manufacturing industry has been rapidly growing in many countries in the world, including Arab countries, resulting in a wide variety of pharmaceutical items for human or animal use [9]. Most pharmaceutical wastewater may contains different amounts of antibiotics, antiviral substances, hormones and anti-serum drugs, as well as non-biodegradable organic intermediates, such as ketones, phenol, etc., which are considered as toxic compounds [2, 7, 10]. Drinking water containing trace pharmaceutical compounds is considered a public health concern since little evidence is recognized about the potential chronic health effects related to long-term ingestion of traces of these compounds [7, 11–13]. Thus, contaminated drinking water with pharmaceutical traces is an emerging issue in environmental toxicology that requires more research and investigation [7]. The number of published articles by a certain country on a certain topic is considered a source of prestige and an acknowledgment of the contribution of that country to the construction of the new concepts [14]. Recently, bibliometric tool is commonly used to examine growth of research in different topics [15–18]. This allows scientists to recognize new lines for the improvement of research [15, 19–25].

However, there are limited studies conducted to investigate research performance pharmaceutical wastewater research [10, 26]. Literature survey showed that no bibliometric studies on pharmaceutical wastewater research have been carried out in Arab countries. Therefore, the aim of the current study was to assess the characteristics of Arab regional productivity in the field of pharmaceutical wastewater research and to compare Arab regional productivity with that of other non-Arab Middle Eastern countries. This might shed light into the status of Arab pharmaceutical wastewater research for environmental or toxicological researchers.

## Methods

Bibliometric analyses are typically performed using one of four common databases, including Web of Knowledge, PubMed, Google Scholar and Scopus [27]. To retrieve documents related to pharmaceutical wastewater, we used the Scopus database on November 21, 2015. Scopus was used to extract our data because it is considered the largest database and indexes a larger number of journals than other databases [27, 28].

For bibliometric analysis, Scopus was searched with the following keywords in the title and abstract to obtain global research output: wastewater*, waste water*, waste-water*, sewage*, pharm*, drug*, hospital* and medic*. The search keywords used were obtained from previous international bibliometric studies on pharmaceutical wastewater [10, 26, 29, 30]. The asterisk (*) was included in the search to minimize number of search keywords since the asterisk is considered a wild card in Scopus search engine. For example, if you entered “medic*”in a search engine, you would get results for medicine, medication, medicinal; which represents all possible word that might start with the five letters (i.e. medic). In this study, the duration for data extraction was set up to 2014. Non-countable documents, such as errata, were excluded from our analysis. For our comparative analysis, documents retrieved were limited to Arab countries listed in Table 1 and for Non-Arab Middle Eastern countries, Israel, Turkey and Iran were investigated for comparative purposes.

**Table 1:** Bibliometric analysis of the 179 documents associated with pharmaceutical wastewater research produced by Arab countries

SCR^a, b^	Countries	Articles (%)	*h*-index	No. of citations	Median citation (Q1-Q3)	Average citation	Collaborations with other countries	Number (%)^c^ of documents with international authors	Most collaborated country	No. of documents with most collaborated country (%)
1^st^	KSA	47 (26.2)	12	396	5 (1-12)	8.43	21	37 (78.7)	Egypt	7 (19.0)
2^nd^	Egypt	38 (21.2)	10	358	2 (0-11.25)	9.42	8	17 (44.7)	KSA	7 (41.2)
3^rd^	Tunisia	17 (9.5)	7	217	6 (1.5-15)	12.76	6	10 (58.8)	France	6 (60.0)
4^th^	Morocco	16 (8.9)	7	197	4.5 (1.25-28.75)	12.3	3	7 (43.8)	France	4 (57.1)
5^th^	Oman	14 (7.8)	4	124	1.5 (0.75-5.75)	8.86	7	9 (64.3)	India	3 (33.3)
6^th^	Algeria	12 (6.7)	5	133	1 (0-16.75)	11.1	2	8 (66.7)	France	6 (75.0)
7^th^	Jordan	11 (6.1)	4	32	3 (0-4)	2.9	3	3 (27.3)	Germany	1 (33.3)
8^th^	UAE	9 (5.0)	4	78	4 (3-14.5)	8.7	1	3 (33.3)	Oman	3 (100.0)
8^th^	Iraq	9 (5.0)	3	37	1 (0.5-9.5)	4.1	3	3 (33.3)	Jordan	1 (33.3)
10^th^	Lebanon	5 (2.8)	3	50	3 (2-21.5)	10	4	3 (60.0)	France	1 (33.3)
10^th^	Qatar	5 (2.8)	2	10	1 (0-4.5)	2	5	3 (60.0)	India	1 (33.3)
12^th^	SAR	3 (1.7)	2	37	9 ^d^	12.3	5	3 (100.0)	Australia	1 (33.3)
12^th^	Palestine	3 (1.7)	3	20	6 ^d^	6.67	3	3 (100.0)	Israel	2 (66.7)
14^th^	Kuwait	2 (1.1)	1	14	1 ^d^	7	14	1 (50.0)	Bangladesh	1 (100.0)
15^th^	LAJ	1 (0.6)	1	1	1 ^d^	1	1	1 (100.0)	Sweden	1 (100.0)

*Abbreviations*: *SCR* Standard Competition Ranking, *Q1–Q3* lower quartile–upper quartile, *KSA* Kingdom of Saudi Arabia, *UAE* United Arab Emirates, *SAR* Syria Arab Republic, *LAJ* Libyan Arab Jamahiriya

^a^Equal countries have the same ranking number, and then a gap is left in the ranking numbers

^b^No data related to pharmaceutical wastewater research were published from Bahrain, Comoros, Djibouti, Mauritania, Somalia, Sudan or Yemen

^c^Percentage of documents with international authors (i.e. from Arab and non-Arab countries) from the total number of documents for each country

^d^Interquartile range was not available due to the small number of articles, which were published from these countries

With the use of Boolean operators “AND”, “OR” and “NOT”, the following search query was reached to estimate the total number of publications related to pharmaceutical wastewater at a global level: (TITLE-ABS (wastewater*) OR TITLE-ABS (“waste water*”) OR TITLE-ABS (waste-water*) OR TITLE-ABS (sewage*) AND TITLE-ABS (pharm*) OR TITLE-ABS (drug*) OR TITLE-ABS (hospital*) OR TITLE-ABS (medic*)) AND PUBYEAR < 2015 AND (EXCLUDE(DOCTYPE, “er”)). Then, this search query was limited to Arab countries or Turkey, Israel or Iran.

### Statistical analyses

The Microsoft® Excel 2007 software program was used for data collection, and statistical software package SPSS 15.0 was used for statistical analysis. Descriptive statistics (i.e. the frequency in count and percentage, sum, average, median and interquartile range) were used. Microsoft Excel 2007 for Windows was used for graphs. All the data were analysed by two investigators (Sa’ed Zyoud and Shaher Zyoud) with regard to several bibliometric indicators, including the date of publications, authorships, journals names with their impact factors (IF), countries of origin, institution, number of citations, collaboration patterns, *h*-index and the type of document. These indicators were considered as quality and quantity indicators and were developed in previous studies in this field [15–18]. International collaborative publications were produced by researchers from multiple countries. Journal Citation Reports (JCR) © Ranking: 2014 was used to obtain IF for journals. The *h*-index, is used to qualify scientific research output for researchers, countries, institutions, etc. [31]. The standard competition ranking (SCR) was used to rank the top 10 most prolific journals and institutions. In addition, SCR was used to rank Arab countries according to their research productivity. Ascending ranking was used, which means that 1^st^ is the highest rank and was considered the most prolific [32].

## Results

Globally, the total number of retrieved documents from Scopus was 6374. Excluding erratum documents resulted in a net total of 6360 documents. By restricting the investigation to the documents that have been published by scholars from the Arab world, their productivity was 179 documents. This figure of productivity represents 2.8 % of total scientific research productivity at global level in fields related to pharmaceutical wastewater. Of the published papers from the Arab region, 84.9 % were original papers and only 5.6 % were reviews. The remainder were other types. The leading countries in pharmaceutical wastewater research were the USA (961; 15.11 %), China (731; 11.49 %) and Germany (566; 8.90 %). Worldwide, Iran ranked 15^th^ while Turkey and Israel ranked 17^th^ and 27^th^ respectively. KSA and Egypt ranked 31^st^ and 35^th^ respectively at the global level.

**Fig. 1:**
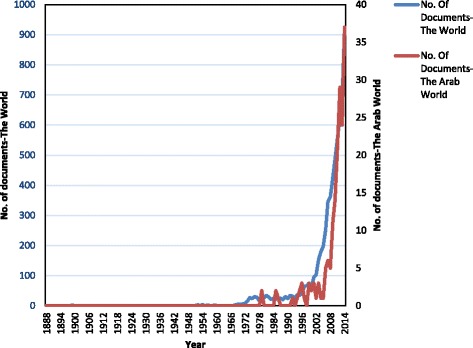
Publications from the Arab world and global pharmaceutical wastewater research

Research activity on pharmaceutical wastewater in Arab countries started in 1979. These research activities grew up in a very modest pattern until the end of the nineteenth century. More than 90.0 % of their output was published after the year 2000 as shown in Fig. 1. The first published articles were in *Radiation Physics and Chemistry* and *Desalination* respectively. The first one, entitled “Status of radiation applications in developing countries”, was by Roushdy, H. M. from the National Centre for Radiation Research, Technology Atomic Energy Authority, Cairo, Egypt [33], and the second one, entitled “A water treatment and reuse program in Riyadh, KSA”, was by Floyd, F.X. from King Faisal Specialist Hospital, Research Centre, Riyadh, KSA [34]. At a global level, the first published work was a conference paper in 1888. It was published in *Public Health* by Corfield, W.H. and entitled “Proceedings of the Society of Medical Officers of Health: On the history of sewage disposal enquiries” [35].

Analysing the most used languages in the published works by researchers from the Arab world shows that 175 documents (98 %) of the total published documents were in English. At a global level, the results of examining the most used languages shows that English is predominant 5614 (88.3 %), followed distantly by Chinese 235 (3.7 %), German 149 (2.3 %) and French 79 (1.2 %).

Table 1 shows the performance indicators resulting from bibliometric analysis for Arab world countries. The results showed that 15 Arab countries have made contributions to pharmaceutical wastewater research. KSA had the largest contribution (47; 26.2 %), followed by Egypt (38; 21.2 %), Tunisia (17; 9.5 %) and Morocco (16; 8.9 %). No data related to pharmaceutical wastewater research were published from Bahrain, Comoros, Djibouti, Mauritania, Somalia, Sudan or Yemen. The total number of citations was 1635, with a mean of 9.13 citations per document. KSA had the highest number of citations (396), followed by Egypt (358), Tunisia (217) and Morocco (197). The retrieved documents attracted an *h*-index of 22. The highest *h*-index was made by KSA researchers with a value of 12, followed by 10 for Egypt and 7 for Tunisia and Morocco for each.

Table 2 demonstrates collaboration patterns with different regions in the world. The study identified 87 (48.6 %) documents with 32 countries resulting from collaboration between the Arab world and the non-Arab world. By examining the results at a regional level, it is noted that Arab researchers collaborated mainly with authors from Western Europe (54; 30.2 %), followed by the Asiatic region (29; 16.2 %) and Northern America (15; 8.4 %). France was, at country level, the most collaborated with country for researchers from the Arab world (n = 20; 11.2 %), followed by Spain (n = 12; 6.7 %) and the USA (n = 12; 6.7 %). The collaboration with Western produced the highest *h*-index with a value of 15 for the published works resulted from collaboration, followed by 10 for the Asiatic region and 6 for Northern America. The highest rates of citation from collaboration with non-Arab countries were 759 for Western Europe, 335 for the Asiatic region and 107 for Northern America.

**Table 2:** Collaboration between Arab countries and non-Arab countries in pharmaceutical wastewater research

Region/Country^a^	No. of Documents (%)
Western Europe	54(30.2)*
France	20(11.2)
Spain	12(6.7)
Germany	8(4.5)
Sweden	3(1.7)
Netherlands	3(1.7)
Italy	3(1.7)
Greece	2(1.1)
Belgium	2(1.1)
Portugal	1(0.6)
Finland	1(0.6)
Norway	1(0.6)
United Kingdom	1(0.6)
Ireland	1(0.6)
Asiatic Region	29(16.2)*
India	9(5.0)
South Korea	6(3.4)
Malaysia	5(2.8)
Pakistan	5(2.8)
China	3(1.7)
Japan	3(1.7)
Singapore	1(0.6)
Sri Lanka	1(0.6)
Bangladesh	1(0.6)
Region/Country	
North America	15(8.4)*
United States	12(6.7)
Canada	5(2.8)
Eastern Europe	3(1.7)*
Romania	2(1.1)
Czech Republic	1(0.6)
Middle East	3(1.7)*
Israel	2(1.1)
Iran	1(0.6)
Latin America	2(1.1)*
Costa Rica	1(0.6)
Brazil	1(0.6)
Pacific Region	1(0.6)*
Australia	1(0.6)
Pacific Region	1(0.6)*
Australia	1(0.6)
Arab-Arab	13(7.3)
Arab-Arab	13(7.3)

^a^The study identified 87(48.6 %)documents with 32 countries in Arab/non-Arab country collaborations

*Total exceeds 48.6 % as data are overlapping due to multi-country collaboration

Table 3 shows the most prevalent areas of interest among the published research from the Arab world. Environmental science was at the top (69; 38.5 %), followed by medicine (38; 21.2 %) and chemistry (37; 20.7 %). The retrieved documents from the global search were published in 142 peer-reviewed journals indexed in Scopus databases, and, for the Arab world, there were 123 peer-reviewed journals. Table 4 presents top journal ranking for publications from Arab countries. *Journal of Hazardous Materials* was at the top with (5; 2.8 %) documents, followed by *Journal of Chromatography A*, *Science of the Total Environment* and *Water Research* with (4; 2.2 %) for each. Nearly most of journals that have been listed in the list of top ten journals had impact factors as referenced in JCR 2014.

**Table 3:** Ranking of areas of interests of the published research in the field of pharmaceutical wastewater within the period of the study

SCR^a^	Areas of Interest	N (%)*
1^st^	Environmental Science	69(38.5)
2^nd^	Medicine	38(21.2)
3^rd^	Chemistry	37(20.7)
4^th^	Biochemistry, Genetics and Molecular Biology	35(19.6)
5^th^	Agricultural and Biological Sciences	22(12.3)
6^th^	Immunology and Microbiology	21(11.7)
7^th^	Chemical Engineering	20(11.2)
8^th^	Materials Science	12(6.7)
9^th^	Engineering	10(5.6)
9^th^	Pharmacology, Toxicology and Pharmaceutics	10(5.6)
10^th^	Earth and Planetary Sciences	9(5.0)

*Abbreviation*: *SCR* Standard Competition Ranking

^a^Equal areas of interest have the same ranking number, and then a gap is left in the ranking numbers

*Total exceeds 100 % as data are overlapping due to multidiscipline interaction

**Table 4:** Ranking of the top 10 journals in which pharmaceutical wastewater related articles were published

SCR^a^	Journal	Frequency	IF (2014)*
1^st^	Journal of Hazardous Materials	5(2.8)	4.529
2^nd^	Journal of Chromatography A	4(2.2)	4.169
2^nd^	Science of the Total Environment	4(2.2)	4.099
2^nd^	Water Research	4(2.2)	5.528
5^th^	Analytical and Bioanalytical Chemistry	3(1.7)	3.436
5^th^	Chemosphere	3(1.7)	3.34
5^th^	Environmental Science and Pollution Research	3(1.7)	2.828
5^th^	World Journal of Microbiology and Biotechnology	3(1.7)	1.779
9^th^	Australian Journal of Basic and Applied Sciences	2(1.1)	NA
9^th^	African Journal of Biotechnology	2(1.1)	NA
9^th^	International Arabic Journal of Antimicrobial Agents	2(1.1)	NA
9^th^	Biological Rhythm Research	2(1.1)	0.919
9^th^	Chemical Engineering Journal	2(1.1)	4.321
9^th^	Journal of Applied Microbiology	2(1.1)	2.479
9^th^	Energy Procedia	2(1.1)	NA
9^th^	Arabian Journal for Science and Engineering	2(1.1)	NA
9^th^	Asian Pacific Journal of Tropical Medicine	2(1.1)	1.062
9^th^	Journal of Environmental Science and Technology	2(1.1)	5.33
9^th^	Journal of Materials and Environmental Science	2(1.1)	NA
9^th^	Talanta	2(1.1)	3.545
9^th^	Water Air and Soil Pollution	2(1.1)	1.554
9^th^	Water Science and Technology	2(1.1)	1.106

*Abbreviations*: *SCR* Standard Competition Ranking, *NA* not available, *IF* impact factor

^a^Equal journals have the same ranking number, and then a gap is left in the ranking numbers

*The impact factor was reported according to journal citation reports (JCR) 2014

Table 5 displays the list of mostly top ten cited documents. Most of these documents, nine documents out of 11 in this list, are articles [36–46]. The citation frequency for the ten most cited documents ranged from 38 to 96 (Table 5). Table 6 is a list of top ten prolific institutions and organizations in pharmaceutical publications from Arab countries. The most prolific institution was *King Saud University* (13; 7.3 %), followed by the *National Research Centre* (10; 7.3 %) and *King Abdulaziz University* (9; 5 %). The list shows that three institutions from KSA, as well as three for Egypt, were in the list of the top ten most prolific institutions.

**Table 5:** Ranking of the top 10 cited articles in Scopus in the field of pharmaceutical wastewater research

SCR^a^	Name of Author and Year of Publication	Title	Type of Document	Journal Name	Times Cited
1^st^	Lavollay et al., 2006 [36]	Clonal dissemination of a CTX-M-15 β-lactamase-producing Eschenchia coli strain in the Paris Area, Tunis, and Bangui	Article	*Antimicrobial Agents and Chemotherapy*	96
2^nd^	Abed et al., 2009 [37]	Applications of cyanobacteria in biotechnology	Review	*Journal of Applied Microbiology*	81
3^rd^	Badawy et al., 2009 [38]	Fenton-biological treatment processes for the removal of some pharmaceuticals from industrial wastewater	Article	*Journal of Hazardous Materials*	59
4^th^	Abdel-El-Haleem, 2003 [39]	Acinetobacter: Environmental and biotechnological applications	Short Survey	*African Journal of Biotechnology*	57
4^th^	Belabbes et al., 1985 [40]	Epidemic non-A, non-B viral hepatitis in Algeria: Strong evidence for its spreading by water	Article	*Journal of Medical Virology*	57
6^th^	Le Roux et al., 2011 [41]	Chloramination of nitrogenous contaminants (pharmaceuticals and pesticides): NDMA and halogenated DBPs formation	Article	*Water Research*	52
7^th^	Yangali-Quintanilla et al., 2010 [42]	Proposing nanofiltration as acceptable barrier for organic contaminants in water reuse	Article	*Journal of Membrane Science*	45
8^th^	Basheer et al., 2010 [43]	Simultaneous extraction of acidic and basic drugs at neutral sample pH: A novel electro-mediated microextraction approach	Article	*Journal of Chromatography A*	41
8^th^	Chafik et al., 2001 [44]	Quality of Moroccan Atlantic coastal waters: Water monitoring and mussel watching	Article	*Aquatic Living Resources*	41
10^th^	Ghauch et al., 2012 [45]	Ibuprofen removal by heated persulfate in aqueous solution: A kinetics study	Article	*Chemical Engineering Journal*	38
10^th^	Reemtsma, 2001 [46]	The use of liquid chromatography-atmospheric pressure ionization-mass spectrometry in water analysis - Part I: Achievements	Article	*TrAC - Trends in Analytical Chemistry*	38

*Abbreviation*: *SCR* Standard Competition Ranking

^a^Equal articles have the same ranking number, and then a gap is left in the ranking numbers

**Table 6:** Ranking of the top 10 most highly productive institutions in the field of pharmaceutical wastewater research during the period of the study

SCR^a^	Name of the Institution	Country	No. of documents (%)
1^st^	King Saud University	KSA	13(7.3)
2^nd^	National Research Centre	Egypt	10(5.6)
3^rd^	King Abdulaziz University	KSA	9(5.0)
4^th^	CSIC - Instituto de Diagnostico Ambiental y Estudios del Agua IDAEA	Spain	7(3.9)
5^th^	Universitat de Girona	Spain	6(3.4)
5^th^	Suez Canal University	Egypt	6(3.4)
5^th^	Faculte des Sciences Semlalia	Morocco	6(3.4)
5^th^	University of Sciences and Technology Houari Boumediene	Algeria	6(3.4)
5^th^	Sultan Qaboos University	Oman	6(3.4)
10^th^	Ain Shams University	Egypt	5(2.8)
10^th^	King Abdullah University of Science and Technology	KSA	5(2.8)

*Abbreviations*: *SCR* Standard Competition Ranking, *KSA* Kingdom of Saudi Arabia

^a^Equal institutions have the same ranking number, and then a gap is left in the ranking numbers

**Fig. 2:**
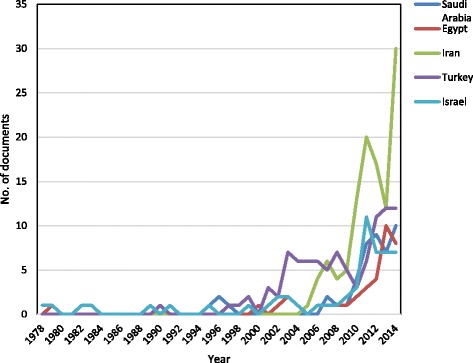
Number of Publications in pharmaceutical wastewater research for the most productive countries – the Arab world and non-Arab Middle Eastern countries

**Fig. 3:**
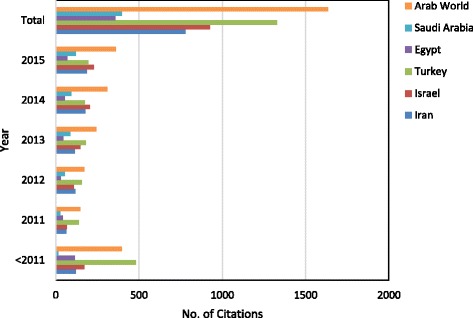
Development of citations for pharmaceutical wastewater research from the Arab world, most productive countries – the Arab world and non-Arab Middle Eastern countries

Table 7 is a comparative analysis between the most productive Arab countries (KSA, Egypt) in one hand and the three non-Arab countries (Iran, Turkey, and Israel) on the other hand. This comparative analysis shows performance indicators that are related to number of published documents, collaboration countries, research productivity from collaboration, citations, *h*-index, most productive institutions and most used journals. Figure 2 illustrates the growth of productivity of pharmaceutical wastewater research from the two most productive Arab countries and the three non-Arab Middle Eastern countries. Comparison of citation pattern among the compared countries is also illustrated in Fig. 3.

**Table 7:** Qualitative and quantitative comparison between the Arab world and the three most productive countries – the Arab world and three major Middle East countries

Region/Country Field	Arab World	KSA	Egypt	Iran	Turkey	Israel
No. of published documents	179	47	38	113	96	54
*h*-index	22	12	10	17	20	19
Total no. of citations	1635	396	358	780	1327	927
Mean no. of citations	9.13	8.43	9.42	6.9	13.83	17.2
Median no. of citations (Q1-Q3)	3(1.0-10.0)	5(1-12)	2(0.0-11.25)	1(0.0-7.0)	4(1.0-14.75)	14(3.0-25.3)
No. of collaboration countries	32	21	8	10	17	14
No. of documents from collaboration (%)	87(48.6 %)	37(78.7 %)	17 (45.0 %)	13(11.5 %)	19(19.8 %)	37(68.5 %)
Most collaborated country (No. of documents-%)	France (20-11.2 %)	Egypt (7-15 %)	KSA (7-18.4 %)	Sweden (4-3.5 %)	Canada (4-4.2 %)	Italy (7-13 %)
Most used language (No. of documents-%)	English (175-98 %)	English (46-98 %)	English (38-100 %)	English (112-99.0 %)	English (92-96 %)	English (54-100 %)
Used mother language (No. of documents-%)	Arabic (0.0-0 %)	Arabic (0.0-0 %)	Arabic (0.0-0 %)	Persian (0-0 %)	Turkish (9-9.4 %)	Hebrew (0.0-0 %)
Most productive institution (No. of documents-%)	King Saud University, KSA (13-7.3 %)	King Saud University, KSA (13-28 %)	National Research Center (10-26.3 %)	Islamic Azad University (21-18.6 %)	Istanbul TeknikUniversitesi (27- 28 %)	Hebrew University of Jerusalem (20-37 %)
Most used journal (No. of documents-%)	*Journal of Hazardous Materials* (5-2.8 %)	*Analytical and Bioanalytical Chemistry* (3-6.4 %)	*Australian Journal of Basic and Applied Sciences, Journal of Hazardous Materials, Talanta, Water Research, Water Science and Technology* (2- 5.3 % for each one)	*Journal of Mazandaran University of Medical Sciences* (13-11.5 %)	*Journal of Hazardous Materials, and Water Science and Technology* (7-7.3 % for each one)	*Water Research* (5-9.3 %)

*Abbreviation*: *KSA* Kingdom of Saudi Arabia

## Discussion

Pharmaceutical wastewater research has recently experienced substantial growth and progress, especially in terms of treatment [47–52]. This can be attributed to the efforts of researchers in environmental, toxicological and pharmaceutical chemistry field in different parts the world. Bibliometric analysis for biomedical or environmental research activity has been carried out to assess scientific research productivity [10, 17, 18, 26, 53, 54]. Recently, several worldwide bibliometric studies on water publications have been carried out [10, 30, 55–59]. Qian et al. [10] used bibliometric indicators to provide a clear picture of research activities and trends in the field of pharmaceutical wastewater treatment at a global level from 1994 to 2013. However, bibliometric analysis was not used earlier to investigate research activities on pharmaceutical wastewater from the Arab region. In this regard, our study is the first to use bibliometric tools to assess publications on pharmaceutical wastewater research in the Arab countries.

In this study, 179 publications on pharmaceutical wastewater produced by Arab countries were extracted from Scopus and analyzed for bibliometric indicators. The result obtained does not include literature published in un-indexed journals. The use of Scopus for data extraction is justifiable given that Scopus is larger than WoS and more accurate the Goggle Scholar [27, 28]. Our study provides a clear and thorough analysis about the research productivity of Arab countries on pharmaceutical wastewater compared with that from other non-Arab Middle Eastern countries. Bibliometric analysis demonstrated that number of publications from Arab countries in pharmaceutical wastewater research is lagging behind that of non-Arab Middle Eastern countries or developed countries. Arab countries are also are in agreement with findings made by several previous studies, especially those in medical fields [60–63]. Relatively poor research productivity from Arab countries in the pharmaceutical wastewater research could be due to lack of professionals and experts in this field or lack of adequate governmental and non-governmental financial support for pharmaceutical wastewater research and its impact on public health in Arab nations [60, 61, 64].

The number of articles in pharmaceutical wastewater research began to increase after 2000, and most of the Arab output was published after the year 2000. Bibliometric studies published previously on worldwide research activity on pharmaceutical wastewater treatment showed a relative increase in the number of published articles with time [10, 26]. The majority of worldwide publications on pharmaceutical wastewater treatment research appeared after 2003 [10]. The increase in research productivity in Arab countries on pharmaceutical wastewater research might be due to an emerging issue in environmental problem manifested as an increase in the number of people from the Arab countries lacking access to safe drinking water. In the Arab countries, due to population growth, the number of people lacking access to good quality drinking water increased by 14 million from 1990 to 2006 [65].

The most interesting finding of this study was that, KSA and Egypt had the largest share of publications on pharmaceutical wastewater. Similar findings were observed in previous bibliometric studies in Arab countries [18, 61, 63, 64, 66, 67]. Previous studies have also shown that the scientific research contribution of KSA and Egypt has notably increased [18, 61, 63, 64, 66, 67]. A possible explanation for this might be that KSA is a wealthy country with increasingly governmental support for research in general. In the case of Egypt, the total number of population and total number of scientists contributed to research output from Egypt compared with other Arab countries. Furthermore, these results are in accord with a recent study indicating that KSA and Egypt had the largest share of publication in Arab countries on public and environmental health [64]. Among institutions, *King Saud University* ranked first in publishing the most articles on pharmaceutical wastewater research from KSA. These results are in agreement with previous findings [63, 68].

The most commonly cited article had 96 times and was published by Lavollay et al. [36] in 2006. This article resulted from collaboration among Tunisia, France and the Central African Republic. This article suggested that plasmid-borne blaCTX-M-15 dissemination between, Tunisia, France and the Central African Republic was due mainly to its residence in an *Escherichia coli* clone with extraordinary propensity for colonization [36]. The second most frequently cited paper was by Abed et al. [37] and published in 2009 as a review article in Oman. This article resulted from collaboration between Oman and Malaysia. The authors reported on the uses of cyanobacteria in industry and suggested an outlook on the challenges and future scenarios in the field of cyanobacterial biotechnology, such as sources of alternative energy, aquaculture and wastewater treatment [37]. The next most cited paper was by Badawy et al. and was published as an original article in Egypt in 2009. The authors demonstrated that the application of the Fenton oxidation process as a pre-treatment would increase the biodegradability of and/or remove pharmaceuticals from wastewater [38].

The present study identified successful collaborations between Arab countries and Western Europe and the Asiatic region. However, Western Europe-based research clearly stands out as the most highly cited and received the highest *h*-index, even though collaboration with Asiatic research groups had a considerable impact and showed an increase in citations and the *h*-index. A previous study showed that researchers with international collaborations might produce higher quality publications, compared with those who do not have international collaborations [64, 69, 70]. Highly cited publications definitely have a positive contribution to the *h*-index of a country, institution or individual author [71]. According to the previous analysis, there is an urgent need for environmental or toxicological researchers from developed countries to widen their research cooperation with developing countries to have more fairness in these developing regions and to achieve the most accurate science.

This study has limitations that are very similar to those of other bibliometric studies published by the principal investigator [15–18]. First, the main limitations of this study were restricting the search only to Scopus, so databases other than Scopus, such as Google Scholar, were not included. Second, using different search terms during the application of the search strategy might yield different results. Third, pharmaceutical wastewater publications that do not contain the related terms as a keyword in their titles or abstracts might not be included in our analysis, so it is possible that not all publications for all pharmaceutical wastewater have been counted.

## Conclusions

This is the first bibliometric analysis assessing the performance Arab productivity in the field of pharmaceutical wastewater research. This study showed that KSA has the largest share of productivity on pharmaceutical wastewater research. Bibliometric analysis demonstrated that research productivity, mainly from Arab countries in pharmaceutical wastewater research, was relatively lagging behind. More research effort is required for Arab countries to catch up with those of non-Arab Middle Easter countries on pharmaceutical wastewater research.

## Abbreviations

IFs, impact factors; JCR, Journal Citation Reports; KSA, Kingdom of Saudi Arabia; SCR, Standard Competition Ranking; SPSS, Statistical Package for Social Sciences; UK, United Kingdom; USA, United States of America
